# Supplementary material for: ASC specks as a single-molecule fluid biomarker of inflammation in neurodegenerative diseases
Source: Nat Commun. 2024 Nov 11;15:9690. doi: 10.1038/s41467-024-53547-0 (PMC11555386; doi:10.1038/s41467-024-53547-0)
Supplement: Supplementary file 1 — Supplementary Information [file 41467_2024_53547_MOESM1_ESM.pdf]

Supplementary Information for "ASC specks as a single-molecule fluid biomarker of inflammation in neurodegenerative diseases"

Evgeniia Lobanova[1,2], Yu P. Zhang[1,2], Derya Emin[1,2], Jack Brelstaff[3], Lakmini Kahanawita[3], Maura Malpetti[3], Annelies Quaegebeur[3], Kathy Triantafilou[4], Martha Triantafilou[4], Henrik Zetterberg[5,6,7,8,9,10], James B. Rowe[3,11], Caroline H. Williams-Gray[3], Clare Elizabeth Bryant[12] and David Klenerman[1,2]

1. Yusuf Hamied Department of Chemistry, University of Cambridge, Lensfield Road, Cambridge CB2 1EW, UK
2. UK Dementia Research Institute at University of Cambridge, Cambridge CB2 0XY, United Kingdom
3. Department of Clinical Neurosciences, University of Cambridge and Cambridge University Hospitals NHS Trust, Cambridge, UK
4. School of Medicine, Division of Infection and Immunity, University Hospital of Wales, Cardiff University, Cardiff, UK
5. Department of Psychiatry and Neurochemistry, Institute of Neuroscience and Physiology, The Sahlgrenska Academy at the University of Gothenburg, Mölndal, Sweden
6. Clinical Neurochemistry Laboratory, Sahlgrenska University Hospital, Mölndal, Sweden
7. Department of Neurodegenerative Disease, UCL Institute of Neurology, Queen Square, London, UK
8. UK Dementia Research Institute at UCL, London, UK
9. Hong Kong Center for Neurodegenerative Diseases, Hong Kong, China
10. Wisconsin Alzheimer's Disease Research Center, University of Wisconsin School of Medicine and Public Health, University of Wisconsin-Madison, Madison, WI, USA
11. Medical Research Council Cognition and Brain Sciences Unit, University of Cambridge, Cambridge, UK
12. Department of Medicine, Box 157, Level 5, Addenbrookes Hospital, University of Cambridge, Cambridge, CB2 0QQ, UK

[#]Correspondence to: dk10012@cam.ac.uk or el519@cam.ac.uk

The supplementary material contains the additional results on IL-1β ELISA from nigericin time course on media from THP-1 macrophages primed with LPS (Fig. 1), cumulative size and shape distributions of ASC specks in the nigericin-stimulated THP-1 cell media and lysates (Fig. 2), single-molecule detection of Aβ and p-tau-AT8 (phosphorylated at positions 202/205) aggregates in AD and control serum (Fig. 3), morphology analysis of super-resolved ASC specks in PD soaked brain compared to controls (Fig. 4) and in the PD (n = 8) compared to AD (n = 9) serum (Fig. 5) as presented in our main manuscript. Also, the comparison in the area and circularity distributions of ASC aggregates acquired with different number of imaging frames in *d*STORM is shown in Fig.6 and also Fig.9. The comparison of *d*STORM performance with versus without the UV laser illumination is shown in Fig. 10. The comparison of *d*STORM performance when acquired in the overlap instead of the overlap camera mode is shown in Fig. 11. The correlation of proinflammatory cytokine panels with ASC specks in AD dementia (n = 20) and HC serum samples (n = 10) as well as in PD (n = 7) and HC (n = 11) serum samples are shown in Figs. 7,8. Analytical precision values of the ASC speck SiMPull assay are shown in Table 1.

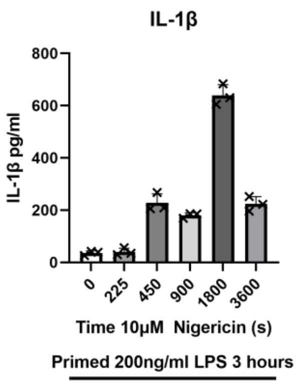

Supplementary Figure 1. IL-1β ELISA from nigericin time course for t = 0, 3.75, 7.5, 15, 30 and 60 mins on media from THP-1 macrophages primed with LPS.

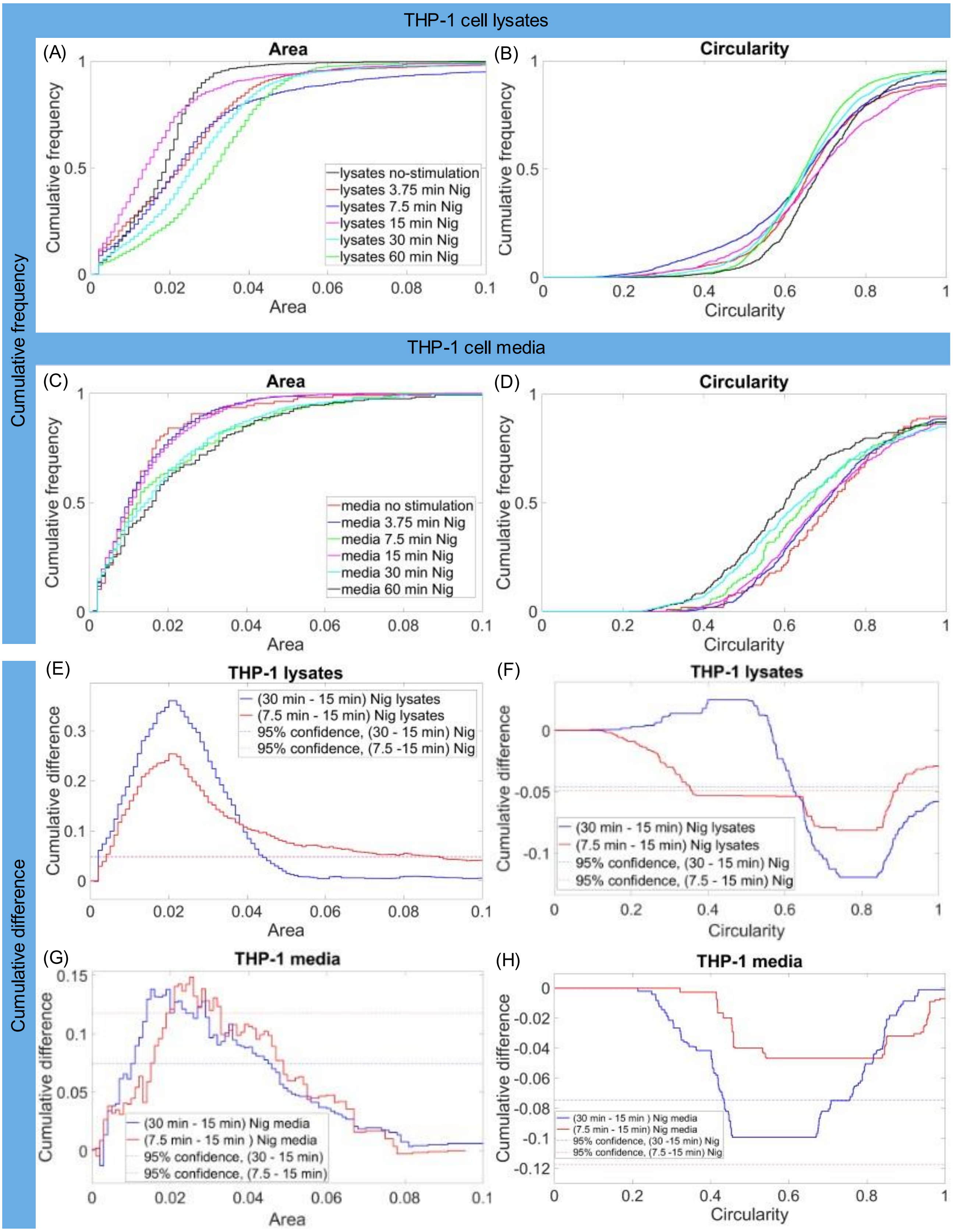

Supplementary Figure 2. **(A-D)** Cumulative (A,C) area and (B,D) circularity distributions of ASC specks for the THP-1 cell media and lysates treated with LPS + Nigericin 10µM for t = 0 (no stimulation), 3.75, 7.5, 15, 30 and 60 mins by *d*STORM. **(E-H)** Difference in cumulative area and shape distributions for THP-1 lysates (**E,F**) and media (**G,H**) between 30 min vs 15 min and 7.5 min vs 15 min nigericin time points retrieved from (**A-D**). The dotted line indicates 95% confidence using the Kolmogorov-Smirnov statistical test.

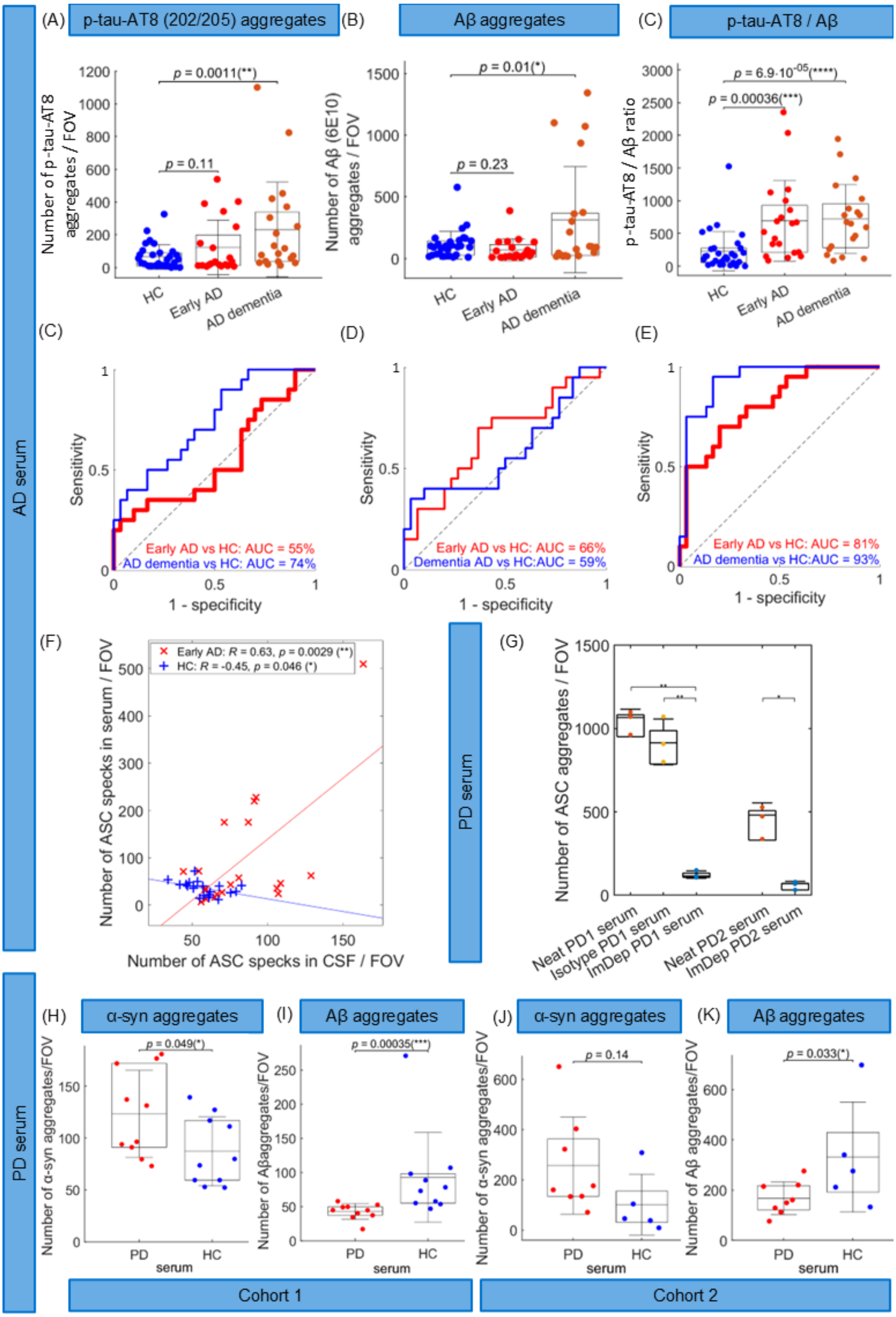

Supplementary Figure 3. (**A-B**) Single-molecule detection of Aβ (6E10) (A) and p-tau phosphorylated at positions 202/205 (AT8) (B) aggregates in early-stage AD (n = 20), late-stage AD (n = 20 AD dementia) and HC (n = 30) serum using SiMPull assay. (**C**) The ratio of the number of p-tau205 to the number of Aβ aggregates in the same samples. The data are shown as mean ± SD, and the lower and upper boundaries of the box indicate the 25th and 75th percentiles, respectively. Permutation (exact) test: * $p < 0.05$, ** $p < 0.01$, *** $p < 0.005$, **** $p < 0.0005$ (**C-E**) ROC curve analysis using single quantity detection of p-tau-AT8 (**C**) and Aβ (**D**) aggregates or the p-tau-AT8/Aβ ratio in serum discriminating controls from people with early-stage AD or controls from people with late-stage AD. (**F**) Correlation in the number of ASC specks in paired serum versus CSF samples from 20 early AD patients and 20 HC participants. (**G**) The number of ASC aggregates detected in two different PD serum samples before (neat) and after immunoprecipitation of ASC protein with an ASC speck-specific (AL177) (ImDep) or an isotype-specific control (Isotype) antibodies. Dots in **(G)** represent independent technical replicates (n = 3). The minimum of 9 fields of view were measured in each replicate. **(H-K)** Quantification of the number of α-syn **(H,J)** and Aβ **(I.K)** aggregates per FOV in PD cohort 1 and PD cohort 2 using SiMPull assay.

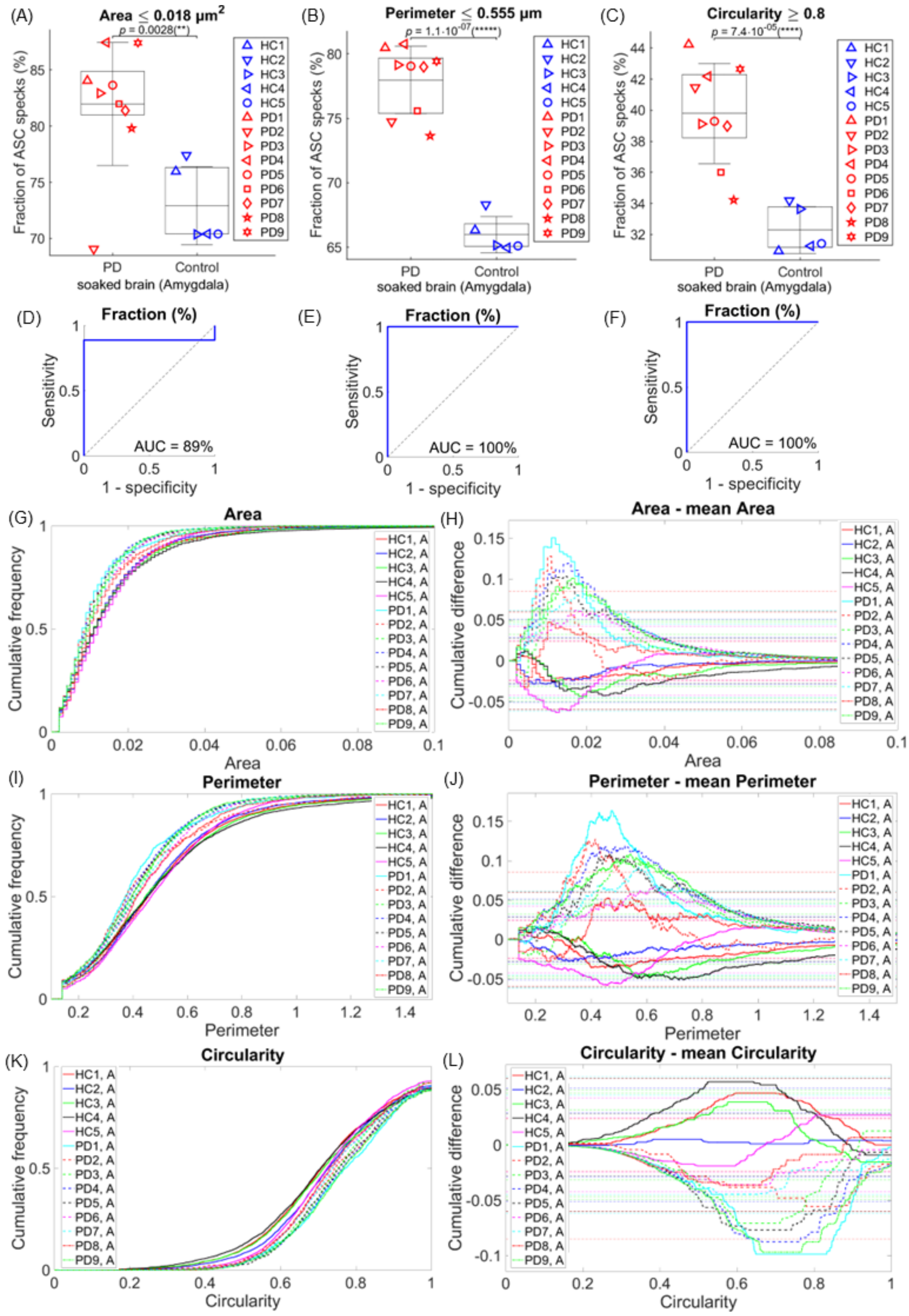

Supplementary Figure 4. Morphology analysis of the super-resolved ASC specks detected in PD soaked brains (n = 9 PD cases) compared to controls (n = 5 non-demented controls) using *d*STORM. (**A-F)** Quantification of super-resolved ASC specks and ROC analysis for PD versus control brain classification by the number of ASC aggregates with area smaller than 0.018 μm^2 (**A**, **D**), with perimeter smaller than 0.555 μm (**B**, **E**) and circularity larger than 0.8 (**C**, **F**) detected in the soaked brain samples. Data in **A-C** are shown as mean ± SD with each circle representing individual patients. The lower and upper boundaries of the box indicate the 25th and 75th percentiles, respectively. The permutation (exact) test: ** $p < 0.01$, **** $p < 0.0005$,***** $p < 0.00005$. (**G, I, K**) Cumulative area (**G**), perimeter (**I**) and circularity (K) distributions for individual patients and controls. (**H, J, L**) Cumulative area (**H**), perimeter (**J**) and circularity (**L**) difference between each individual and mean distribution retrieved from (**G, I, K**). The dotted line indicates 99% confidence using the Kolmogorov-Smirnov statistical test. Legend indicate patient numbers.

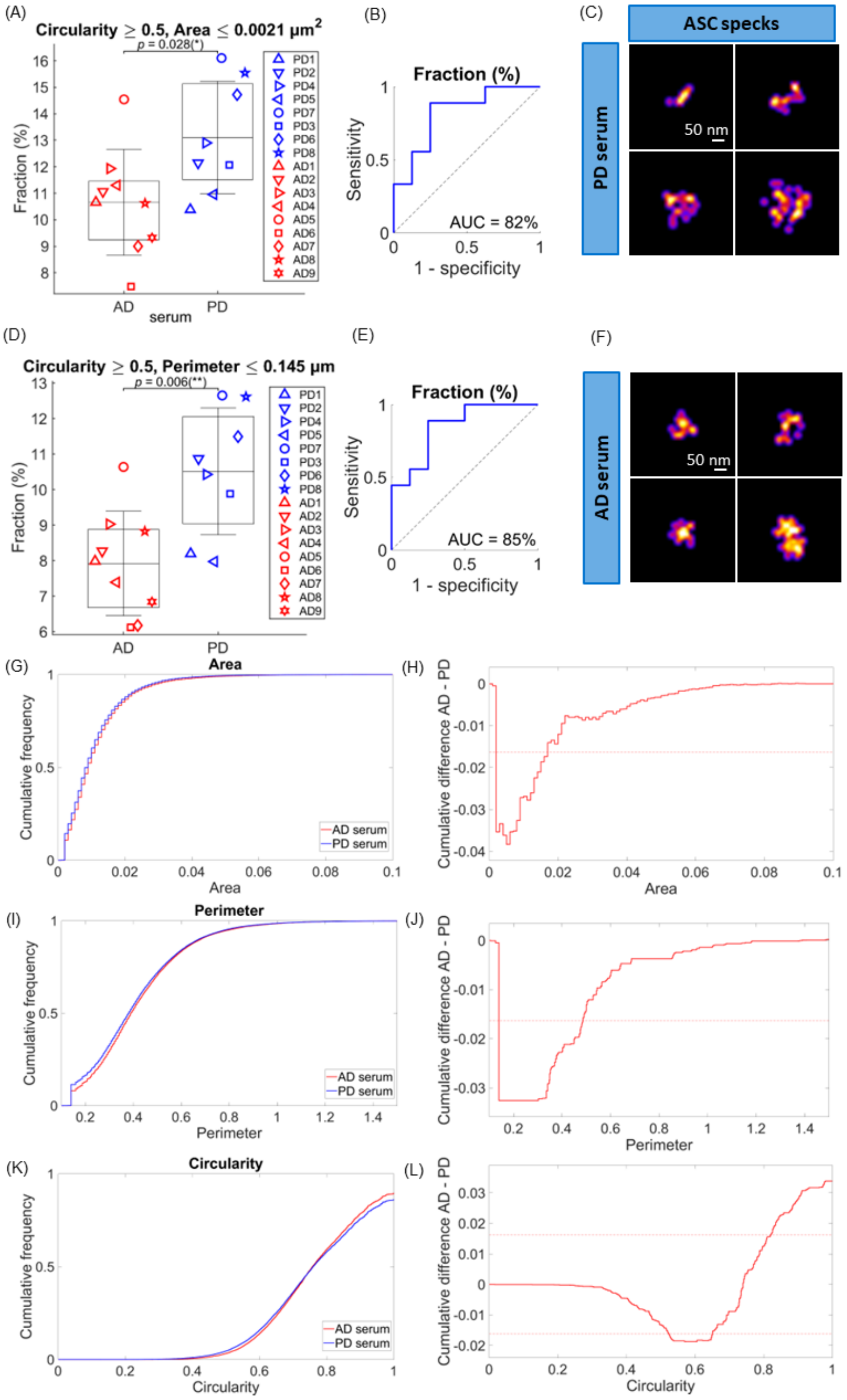

Supplementary Figure 5. Morphology analysis of ASC specks detected in the PD (n = 8) vs AD (n = 9) serum using *d*STORM. **(A,D)** Comparison in the fraction of individual ASC aggregates that are smaller (area ≤ 0.018 μm^2 in **A** or perimeter ≤ 0.145 μm^2 in **D**) and rounder (circularity ≥ 0.5) than the defined threshold allowed to distinguish ASC specks that are more present in PD than AD serum. (**B,E**) ROC curve analysis of the identified PD over AD phenotype of ASC specks distinguishing two diagnosis (AUC = 82-85%). (**C, F**) Examples of super-resolved ASC aggregates in PD (**C**) and AD (**F**) serum samples. Cumulative area (**G**), perimeter (**I**) and circularity (**K**) distributions of ASC specks for PD vs AD serum. Difference between PD and AD cumulative area (**H**), perimeter (**J**) and circularity (**L**) distributions retrieved from **G**, **I** and **K**. The dotted line indicates 99% confidence using the Kolmogorov-Smirnov statistical test.

Is 8000 frames enough to accurately measure size and shape of ASC speck?

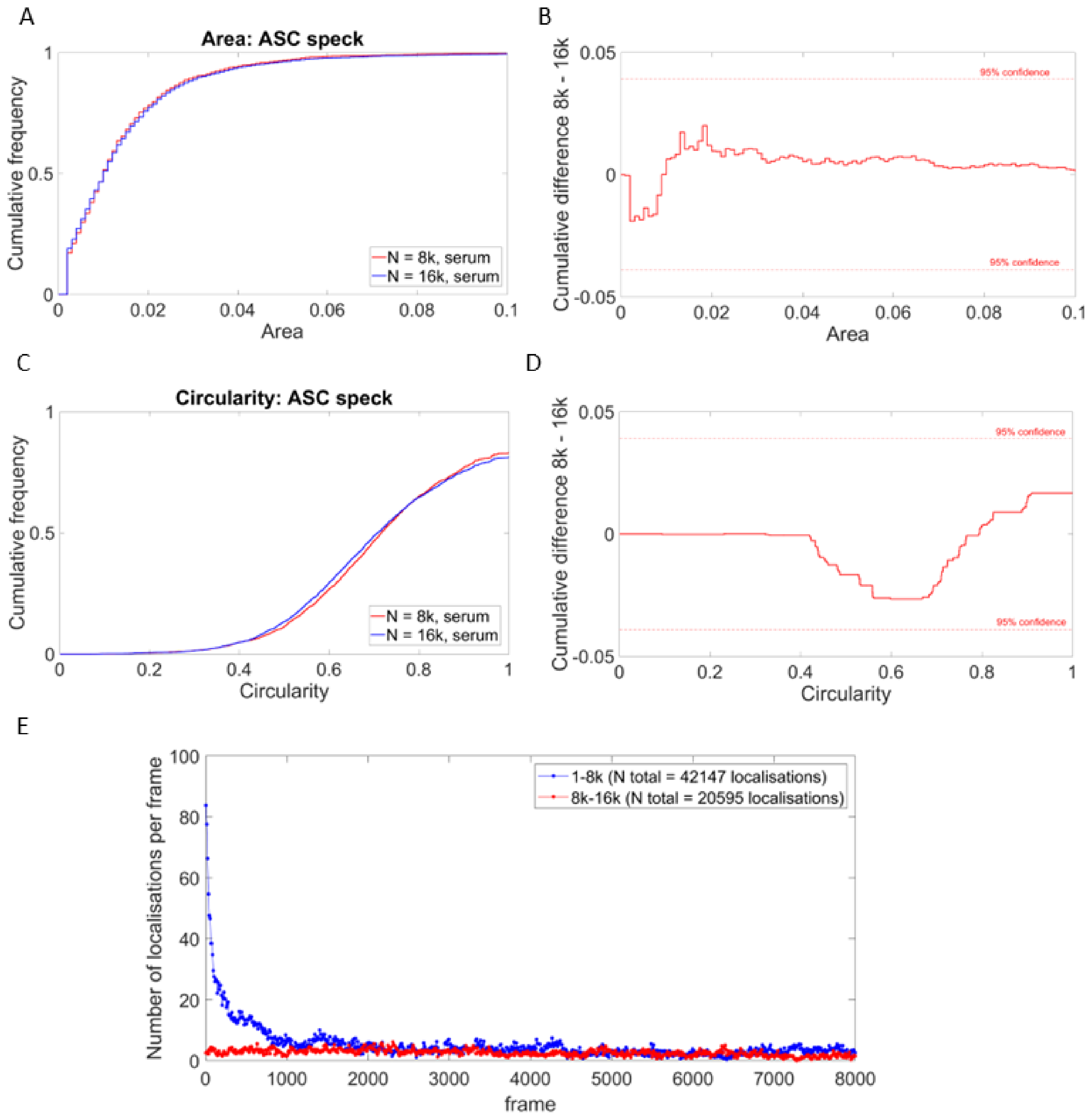

Supplementary Figure 6. Head-to-head comparison of the size and shape of ASC specks in serum extracted from *d*STORM images using 8k versus 16k frames. Cumulative area (**A**) and circularity (**C**) distributions of ASC specks in the same PD serum sample imaged using 8k vs 16k frames. The distributions were calculated from 5 different field of views (images). No significant difference (within a 95% confidence interval) in the size (**B**) and shape (**D**) distributions of ASC aggregates in serum were found when imaged with 16k frames instead of 8k frames. **(E)** Comparison of the number of localisations from 1-8k (blue) and from 8k-16k (red) frames. Red curve is shifted along the x-axis for 8000 frames to the left (x limits = [8k,16k]) for clarity. No photo-bleaching of all fluorophores was observed after 8k frames, as evidenced by sufficient number of localisations detected from 8k-16k frames.

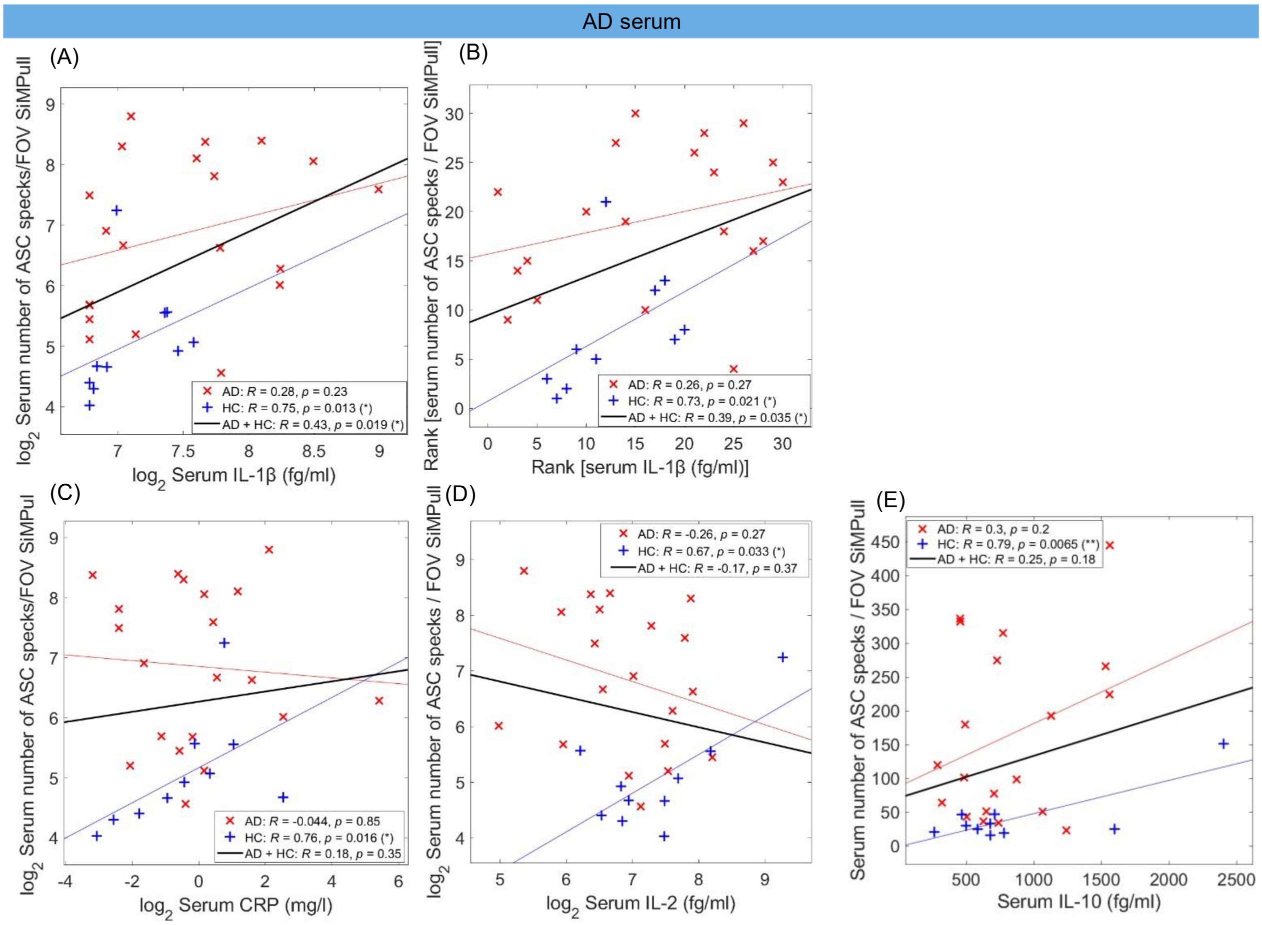

Supplementary Figure 7. Correlation of the ASC speck with the cytokines IL-1β, IFN-γ, IL-2, IL-4, IL-6, IL-10, IL-12p70, IL-17A, TNF-α in AD and HC serum.

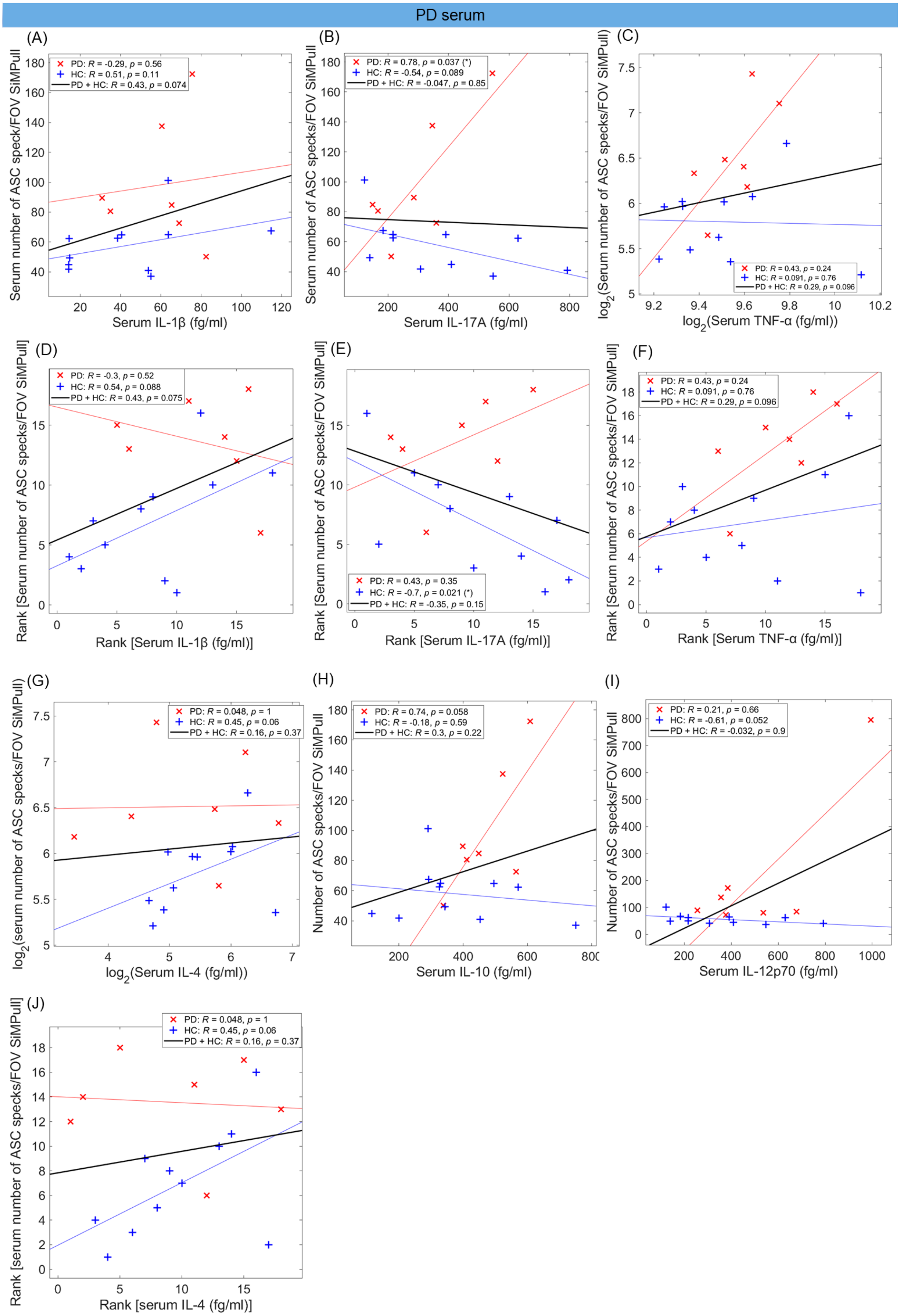

Supplementary Figure 8. Correlation of the ASC speck with the cytokines IL-1β, IFN-γ, IL-2, IL-4, IL-6, IL-10, IL-12p70, IL-17A, TNF-α in AD and HC serum.

Area distribution of ASC aggregates in a serum sample as a function of the number of imaging frames

A

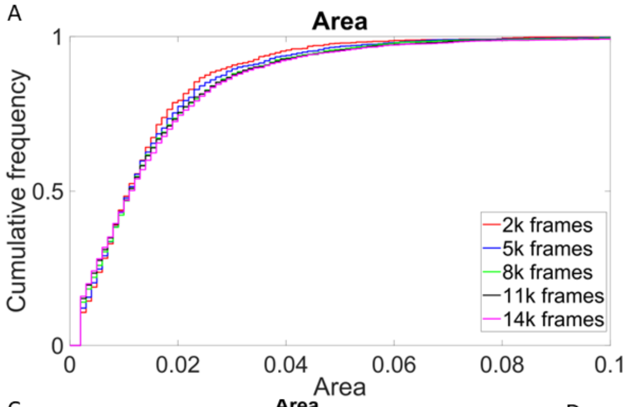

B

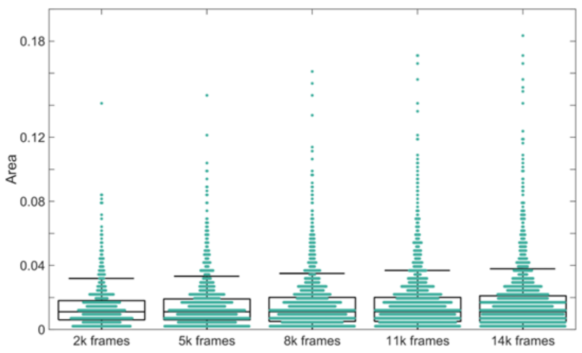

C

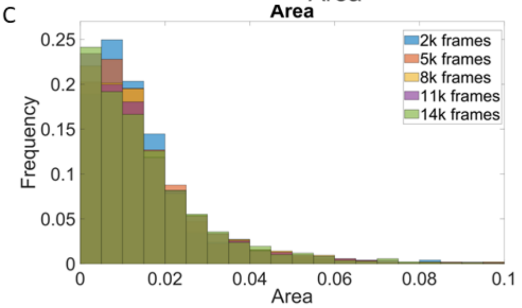

D

Number of imaging frames	Mean	STD	Median	CI_25	CI_75	IQR
2k frames	0.0146	0.0173	0.011	0.006	0.018	0.012
5k frames	0.0153	0.0179	0.011	0.006	0.019	0.013
**8k frames**	**0.016**	**0.019**	**0.011**	**0.005**	**0.02**	**0.015**
**11k frames**	**0.0163**	**0.0206**	**0.011**	**0.005**	**0.02**	**0.015**
**14k frames**	**0.0165**	**0.0213**	**0.011**	**0.005**	**0.021**	**0.016**

Circularity distribution of ASC aggregates in a serum sample as a function of the number of imaging frames

E

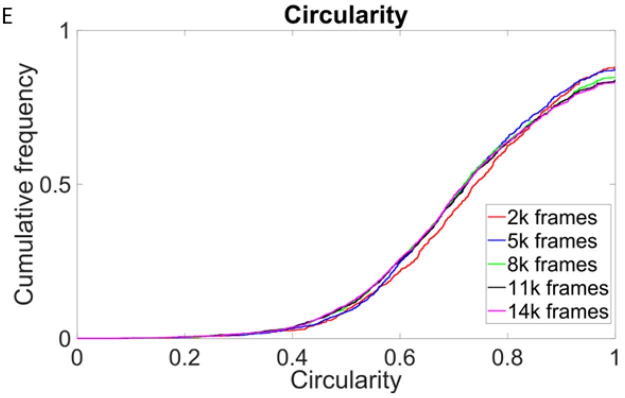

F

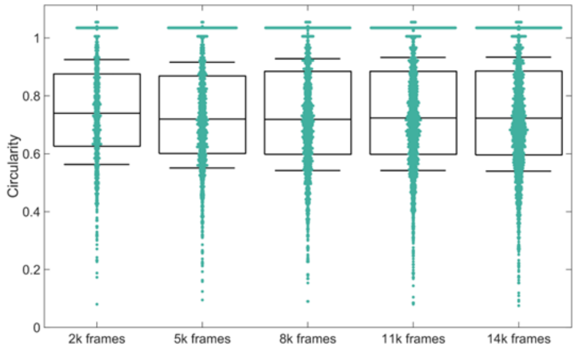

G

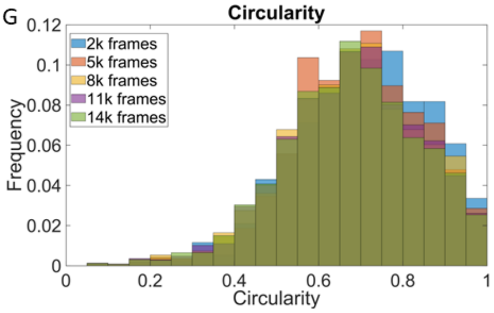

H

Number of imaging frames	Mean	STD	Median	CI_25	CI_75	IQR
2k frames	0.744	0.181	0.74	0.626	0.876	0.25
5k frames	0.734	0.183	0.72	0.601	0.869	0.268
**8k frames**	**0.735**	**0.193**	**0.719**	**0.598**	**0.885**	**0.287**
**11k frames**	**0.738**	**0.196**	**0.724**	**0.598**	**0.885**	**0.287**
**14k frames**	**0.737**	**0.197**	**0.723**	**0.596**	**0.886**	**0.29**

Supplementary Figure 9. The spread and differences in the area (A-D) and circularity (E-H) distributions of serum ASC aggregates acquired with 2k, 5k, 8k, 11k and 14k frames in *d*STORM.

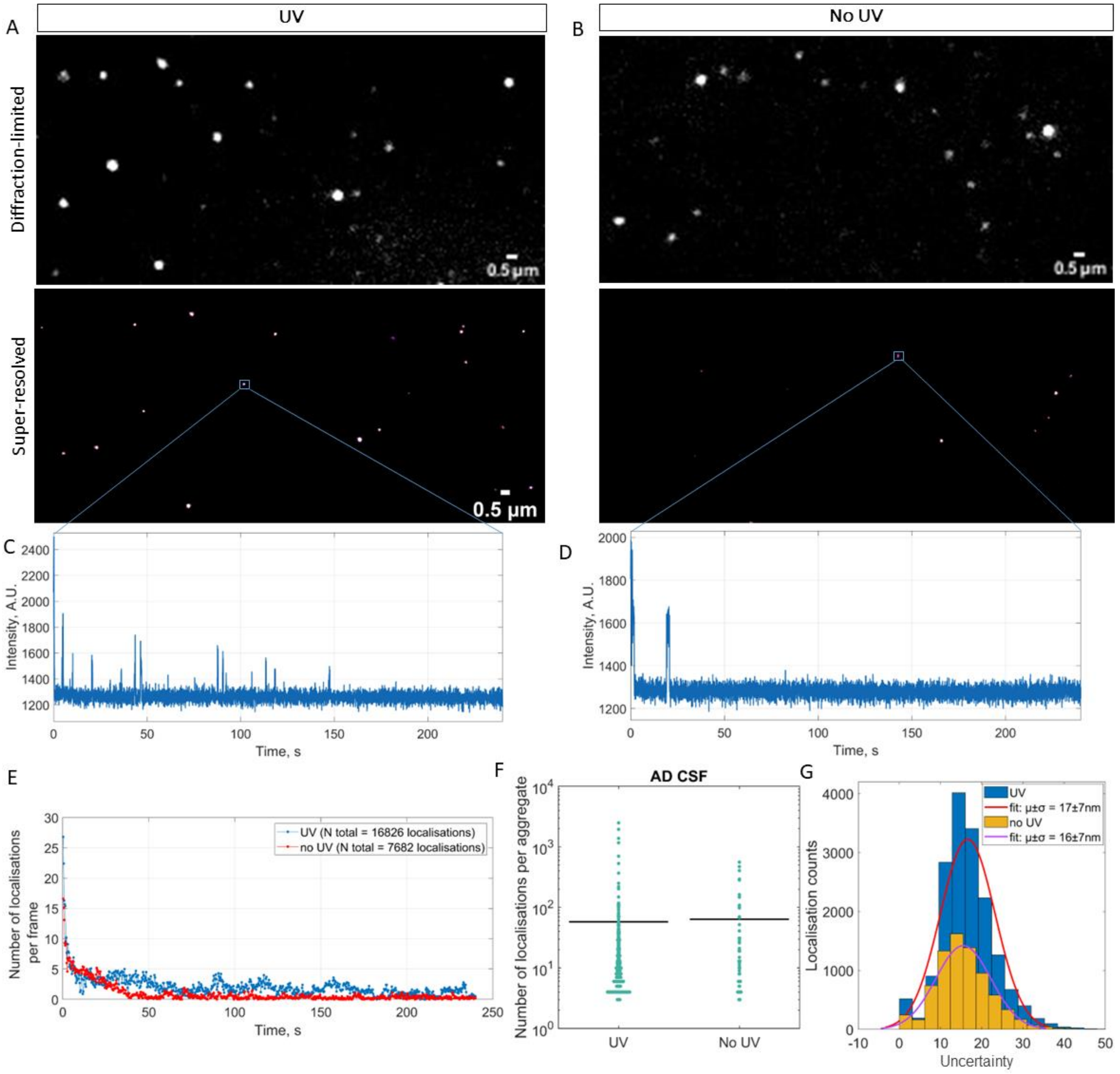

Supplementary Figure 10. *d*STORM imaging with UV versus without UV laser illumination: (A-B) Diffraction limited and super-resolved images (size = 27 x 13 μm) of ASC aggregates in AD CSF (scale bar = 0.5 μm); **(C-D)** Intensity of single-molecule localisations as a function of time and **(E)** the number of localisations as a function of imaging time (t = 30 ms/frame * frame number, N total = 8000 frames) measured with versus without the UV illumination; **(F)** Scatter plot of the number of localisations per ASC aggregate (horizontal line indicates the mean value); **(G)** Lateral uncertainty when measured with UV versus no UV.

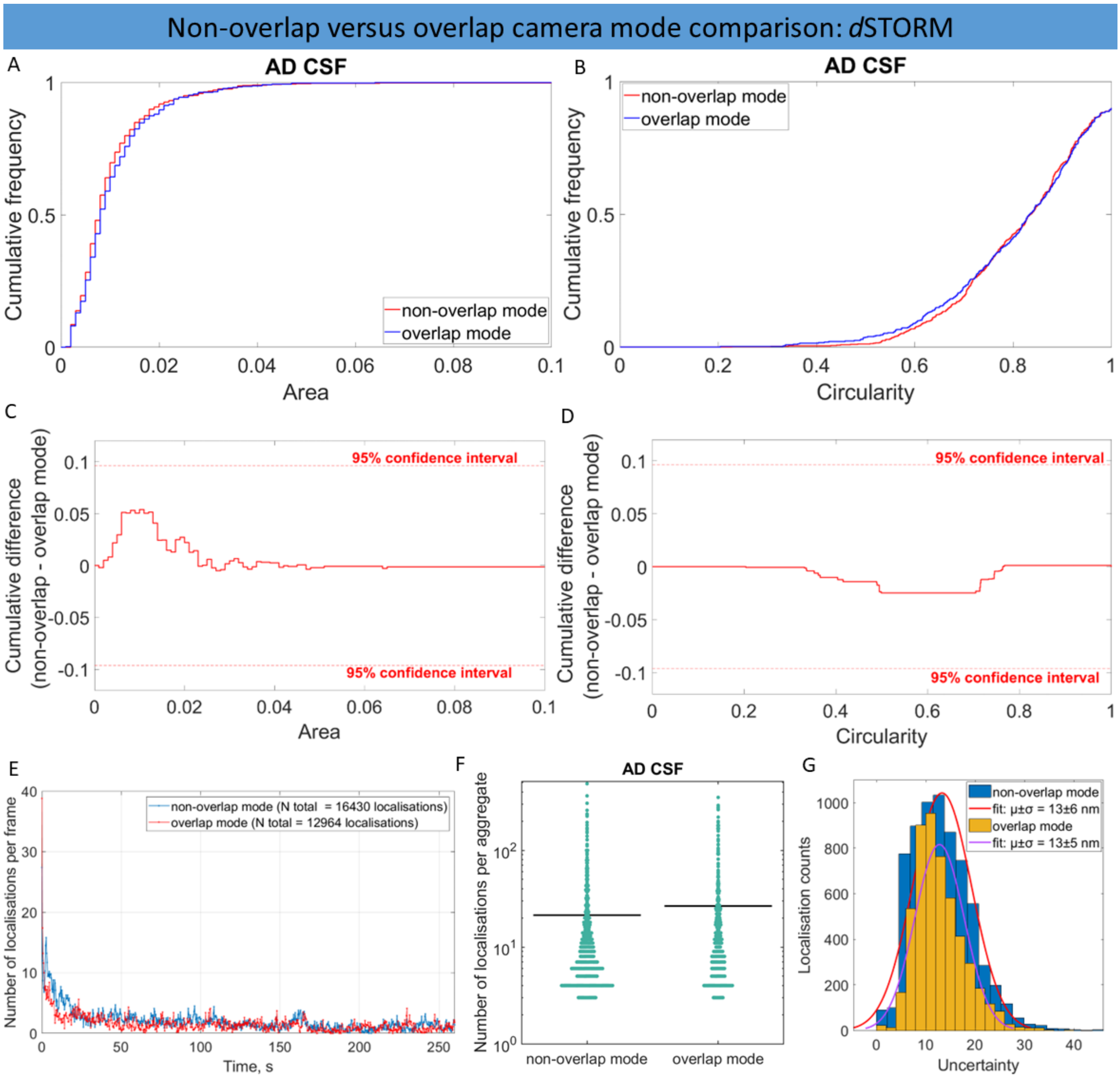

Supplementary Figure 11. *d*STORM acquired in the non-overlap versus overlap (simultaneous exposure-readout) camera mode: N total = 8000 frames, exposure time = 33 ms for both modes. Cumulative area **(A)** and circularity **(B)** distributions of ASC specks in the same AD CSF sample imaged in the non-overlap and overlap mode. The distributions were calculated from 4 different field of views (images). No significant difference (within a 95% confidence interval) in the size **(C)** and shape **(D)** distributions of ASC aggregates in CSF were found when imaged in the overlap instead of non-overlap mode; **(E)** the number of localisations as a function of effective imaging time (t = 33 ms/frame * frame number, N total = 8000 frames) measured in the non-overlap compared to overlap mode; **(F)** Scatter plot of the number of localisations per ASC aggregate (horizontal line indicates the mean value); **(G)** Lateral uncertainty when measured in the non-overlap versus overlap mode.

Supplementary Table 1. ASC speck assay precision

	Serum samples	ASC standards
Average Intra-assay %CV	7	12
Average Inter-assay %CV	15	14
