# Supplementary material for: ASC specks as a single-molecule fluid biomarker of inflammation in neurodegenerative diseases
Source: Nat Commun. 2024 Nov 11;15:9690. doi: 10.1038/s41467-024-53547-0 (PMC11555386; doi:10.1038/s41467-024-53547-0)
Supplement: Supplementary file 2 — Reporting Summary [file 41467_2024_53547_MOESM2_ESM.pdf]

nature portfolio

Corresponding author(s): Evgeniia Lobanova, David Klenerman

Last updated by author(s): Sep 29, 2024

Reporting Summary

Nature Portfolio wishes to improve the reproducibility of the work that we publish. This form provides structure for consistency and transparency in reporting. For further information on Nature Portfolio policies, see our Editorial Policies and the Editorial Policy Checklist.

Statistics

For all statistical analyses, confirm that the following items are present in the figure legend, table legend, main text, or Methods section.

n/a	Confirmed	
☐	☒	The exact sample size (*n*) for each experimental group/condition, given as a discrete number and unit of measurement
☐	☒	A statement on whether measurements were taken from distinct samples or whether the same sample was measured repeatedly
☐	☒	The statistical test(s) used AND whether they are one- or two-sided<br>*Only common tests should be described solely by name; describe more complex techniques in the Methods section.*
☐	☒	A description of all covariates tested
☐	☒	A description of any assumptions or corrections, such as tests of normality and adjustment for multiple comparisons
☐	☒	A full description of the statistical parameters including central tendency (e.g. means) or other basic estimates (e.g. regression coefficient) AND variation (e.g. standard deviation) or associated estimates of uncertainty (e.g. confidence intervals)
☐	☒	For null hypothesis testing, the test statistic (e.g. *F*, *t*, *r*) with confidence intervals, effect sizes, degrees of freedom and *P* value noted<br>*Give P values as exact values whenever suitable.*
☒	☐	For Bayesian analysis, information on the choice of priors and Markov chain Monte Carlo settings
☐	☒	For hierarchical and complex designs, identification of the appropriate level for tests and full reporting of outcomes
☐	☒	Estimates of effect sizes (e.g. Cohen's *d*, Pearson's *r*), indicating how they were calculated

Our web collection on statistics for biologists contains articles on many of the points above.

Software and code

Policy information about availability of computer code

Data collection	MicroManager (Micro-Manager 2.0.0) open source software was used for data collection (imaging).
Data analysis	Data were analysed using MATLAB (R2020b). The diffraction-limited data were analysed using in-house software called Path-Connected Aggregate Recognition (PCAR) which is freely available from https://github.com/LobanovaEG-LobanovSV/PCAR. dSTORM data were analysed using established ImageJ plug-ins. The drift correction, image reconstruction, and morphology analysis was performed by mean shift algorithm, ThunderSTORM (version 1.3), and morphology library, respectively (see the Methods section for details). A custom-written Matlab code was used to integrate mentioned plug-ins and automate data analysing. The code is available from https://github.com/YPZ858/Super-res-code/issues/1.

For manuscripts utilizing custom algorithms or software that are central to the research but not yet described in published literature, software must be made available to editors and reviewers. We strongly encourage code deposition in a community repository (e.g. GitHub). See the Nature Portfolio guidelines for submitting code & software for further information.

Data

Policy information about availability of data

All manuscripts must include a data availability statement. This statement should provide the following information, where applicable:

- Accession codes, unique identifiers, or web links for publicly available datasets
- A description of any restrictions on data availability
- For clinical datasets or third party data, please ensure that the statement adheres to our policy

Source data are provided with this paper. All other data are available from the corresponding author on request.
The code for the diffraction-limited data analysis is publicly available at https://github.com/LobanovaEG-LobanovSV/PCAR
The code for the super-resolution data analysis is publicly available at https://github.com/YPZ858/Super-res-code/issues/1

Research involving human participants, their data, or biological material

Policy information about studies with human participants or human data. See also policy information about sex, gender (identity/presentation), and sexual orientation and race, ethnicity and racism.

Reporting on sex and gender	The data on sex of all study participants were collected based on self-report. Participants without neurological disease were statistically sex-matched to the patients with disease (see Table 1,2 for details). The gender data have not been collected. No sex-based analyses have been performed due to low sample size (N < 10 per group) as per power calculations below.
Reporting on race, ethnicity, or other socially relevant groupings	We have not used the race, ethnicity, or other socially relevant variables in our study.
Population characteristics	Participants with Parkinson's disease were assessed using the Movement Disorder Society Unified Parkinson's Disease Rating Scale (MDS-UPDRS), and the Addenbrooke's Cognitive Examination (ACE-III or ACE-R). Parkinson's disease stage was determined using the Hoehn and Yahr scale (Table 1). Patients with Alzheimer's disease (AD, including the prodromal state of mild cognitive impairment) and participants without neurological disease were recruited in the GOLDeN Study (Genetics of Leucopathology, Dementia and Neurodegeneration) at the Cambridge University Hospitals NHS Trust)(Table 2). Participants with mild cognitive impairment were followed clinically to confirm progression and/or have biomarker evidence of underlying Alzheimer's disease pathology. The participants will also underwent cognitive testing such as the ACE-R and Mini–mental state examination (MMSE), with the MMSE score < 24 (30) taken as an indicator of dementia. Patient samples from the GOLDeN Study analysed in this study were collected in the established dementia phase (n = 20 cases, MMSE score = 18.4 ± 6.8). Patients with early-stage Alzheimer's disease included in this study (n = 20 cases) were recruited from memory clinics in Sweden where they sought medical advice for the first time and were diagnosed with AD based on a positive AD CSF biomarker profile, defined as a CSF Aβ42/p-tau181 ratio < 10.25 (as measured by Lumipulse G600II, Fujirebio). Paired serum and CSF samples from these early-stage patients were used.
Recruitment	Patients with idiopathic Parkinson's disease were recruited through the Parkinson's Disease Research Clinic at the John Van Geest Centre for Brain Repair, University of Cambridge/Cambridge University Hospitals NHS Trust, UK. Age and sex-matched participants without neurological disease were also recruited from the NIHR Cambridge Bioresource (http://www.cambridgebioresource.org.uk). Patients with Alzheimer's disease (AD, including the prodromal state of mild cognitive impairment) and participants without neurological disease were recruited in the GOLDeN Study (Genetics of Leucopathology, Dementia and Neurodegeneration) at the Cambridge University Hospitals NHS Trust. Patients with recent diagnosis of Alzheimer's disease included in this study (n = 20 cases) were recruited from memory clinics in Sweden.We selected a homogeneous group of PD/AD patients (all early stage for PD or the same disease stage for AD) together with age and gender-matched controls where possible.
Ethics oversight	Blood and CSF samples - Ethical approval was obtained from the East of England - Essex Research Ethics Committee (16/EE/0445), and East of England - Cambridge Central Research Ethics Committee (03/303, and 15/EE/0270). Informed consents was provided by all participants. The study protocol was also approved by the regional ethics committee at the University of Gothenburg.<br><br>Post-mortem brain donors - the post-mortem work was approved by the London—Bloomsbury Research Ethics Committee; 16/LO/0508. The brain samples have been voluntarily donated without any compensation and with the ethics allowing to release the associated anonymised, non-identifiable clinical and pathological metadata.

Note that full information on the approval of the study protocol must also be provided in the manuscript.

Field-specific reporting

Please select the one below that is the best fit for your research. If you are not sure, read the appropriate sections before making your selection.

☒ Life sciences ☐ Behavioural & social sciences ☐ Ecological, evolutionary & environmental sciences

For a reference copy of the document with all sections, see nature.com/documents/nr-reporting-summary-flat.pdf

Life sciences study design

All studies must disclose on these points even when the disclosure is negative.

Sample size	In our previous studies 10 diseased by 10 control serum samples were sufficient to observe statistically significant differences (p = 4.3 x 10-5) in the levels of studied protein aggregates using our single-molecule imaging technique giving a large effect size (Cohen's d) of 2.14 Therefore, we validated our developed biomarkers in this study using the sample size N >= 10 per diagnostic group. This analysis allowed to achieve power of 99% for detecting case-control differences at a significance level of p = 0.05.
Data exclusions	No data were excluded
Replication	All experiments were reliably reproduced with three independent replicates, detailed statistical analysis is reported.
Randomization	The samples were experimentally analysed randomly. The samples with different diagnostic groups were added to the assay plate in the random order minimising the grouping effect.
Blinding	The samples were analysed in the blinded manner.

Reporting for specific materials, systems and methods

We require information from authors about some types of materials, experimental systems and methods used in many studies. Here, indicate whether each material, system or method listed is relevant to your study. If you are not sure if a list item applies to your research, read the appropriate section before selecting a response.

Materials & experimental systems

n/a	Involved in the study	
☐	☒	Antibodies
☐	☒	Eukaryotic cell lines
☒	☐	Palaeontology and archaeology
☒	☐	Animals and other organisms
☒	☐	Clinical data
☒	☐	Dual use research of concern
☒	☐	Plants

Methods

n/a	Involved in the study	
☒	☐	ChIP-seq
☒	☐	Flow cytometry
☒	☐	MRI-based neuroimaging

Antibodies

Antibodies used

-unconjugated azide-free ASC antibody: AL177, AG-25B-0006-C100, AdipoGen;
- unconjugated α-syn antibody: 211, sc-12767, Santa Cruz;
- biotinylated Aβ antibody: 6E10, 803007, Biolegend;
- biotinylated p-tau: AT8, MN1020B, ThermoFisher;
- Alexa Fluor 647 6E10 Antibody, 803021, Biolegend;
- Alexa Fluor 647 211 Antibody, sc-12767 AF647, Santa Cruz;
-The Alexa 647 conjugated anti-ASC and AT8 antibodies were generated using Zip Alexa Fluor™ Rapid Antibody Labeling Kit (Cat. No. Z11235) and the biotinylated anti-ASC and 211 antibodies were generated using a Pierce™ FITC Antibody Labeling Kit (Cat. No. 53027).

All biotinylated antibodies were diluted from the stock to 10nM and detection antobodies to 5nM. 1mg/ml of antibody is approx. 6667nM.

Validation

-anti-ASC pAb (AL177) antibody was validated in the western blot analysis of ASC expression in human (293-T, Jurkat, Raj, Ramos, BJAB, THP-1, U937, K562, Raw, HeLa) and mouse (EL-4, A20) cell lines by the manufacturer (AdipoGen) and high-impact papers [1-3].

-anti-α-syn 211 antibody was validated in the Western blot analysis of α-synuclein expression in human brain and human fetal brain tissue extracts by the manufacturer (Santa Cruz).

-anti-Aβ 6E10 antibody was validated in the Western blot analysis of human brain lysates, human Aβ1-40 and Aβ1-42 peptides by the manufacturer (Biolegend).

-anti-p-tau AT8 antibody was verified by Cell treatment to ensure that the antibody binds to the antigen stated (by the manufacturer ThermoFisher).

References:
1. Franklin, B.S. et al.The adaptor ASC has extracellular and 'prionoid' activities that propagate inflammation. Nat. Immunol. 15, 727 (2014);
2. Heneka, M.T. et al. NLRP3 is activated in Alzheimer's disease and contributes to pathology in APP/PS1 mice. Nature 493, 674 (2013)

3. Venegas, C. et al. Microglia-derived ASC specks cross-seed amyloid-β in Alzheimer's disease. Nature 552, 355–361 (2017).

Eukaryotic cell lines

Policy information about cell lines and Sex and Gender in Research

Cell line source(s)	The immortalized human monocyte cell line THP-1 were aquired from American Type Culture Collection.
Authentication	THP-1 cell lines were authenticated via STR analysis.
Mycoplasma contamination	Mycoplasma contamination was excluded through PCR-based and luminescence-based mycoplasma assays.
Commonly misidentified lines (See ICLAC register)	Cell lines used are not in ICLAC.
